# Guidance to develop a multidisciplinary, international, pediatric registry: a systematic review

**DOI:** 10.1186/s13023-023-02901-4

**Published:** 2023-09-21

**Authors:** S. Ombashi, P. A. J. van der Goes, S. L. Versnel, R. H. Khonsari, A. E. Mink van der Molen

**Affiliations:** 1 Department of Plastic, Reconstructive and Hand Surgery, Erasmus Medical Center, Rotterdam, The Netherlands; 2 Service de Chirurgie Maxillofaciale et Chirurgie Plastique, Hôpital Necker - Enfants Malades, Assistance Publique - Hôpitaux de Paris; Faculté de Médecine, Université Paris Cité, Paris, France; 3 Department of Plastic, Reconstructive and Hand Surgery, University Medical Center, Utrecht, The Netherlands; 4 Scientific Committee, ERN CRANIO, Rotterdam, The Netherlands

## Abstract

**Aim:**

The European Reference Network for craniofacial anomalies and ear, nose and throat disorders (ERN-CRANIO) aims to improve craniofacial care on a European scale. Within ERN-CRANIO, the cleft lip and palate (CL/P) work stream seeks to ameliorate health outcomes for patients with CL/P. This work stream acknowledged the need for a European wide registry for comparable outcome measures and therapy endpoints to achieve this goal. This review aimed to provide a scientific basis for the conceptualization of this registry by studying previous registry initiatives.

**Methods:**

This review performed thematic analysis on twenty-four articles through narrative synthesis. An iterative process was used to identify key-themes required for prolonged registry success.

**Results:**

Analysis of the literature resulted in twenty-one distinct headings including quantitative and qualitative data. Quantitative data including registry characteristics were visualized in a table. The analysis of qualitative data resulted in the identification of fourteen key-themes, which have been summarized and visualized in a guidance.

**Conclusion:**

This review has successfully identified key-themes required for the development of an international, multidisciplinary, pediatric registry for pan-European cleft care. The guidance provided by this review applies to the goals of ERN-CRANIO, but can be used by any initiative developing a registry.

**Supplementary Information:**

The online version contains supplementary material available at 10.1186/s13023-023-02901-4.

## Key points

- Twenty-four previous multidisciplinary, pediatric registry initiatives were analyzed using thematic analysis in an iterative process to identify key-themes required for registry success.
- Fourteen key-themes for the successful development of an international, multidisciplinary, pediatric registry were identified.
- This study provides the scientific basis for the development of the ERN-CRANIO CL/P registry, however this study is of use to any initiative seeking to develop a registry.

**Supplementary Information:**

The online version contains supplementary material available at 10.1186/s13023-023-02901-4.

## Introduction

The European Reference Network for Craniofacial anomalies and ear, nose, and throat disorders (ERN CRANIO) is a Europe-wide initiative to improve craniofacial care [1]. ERN CRANIO seeks to achieve health goals unattainable by a single country, by combining the disease-specific expertise, knowledge, and resources of the ERN CRANIO member healthcare centers. Within ERN CRANIO, workstreams for distinctive craniofacial diagnosis were created. One of these workstreams is the cleft lip and palate and orodental anomalies workstream (CL/P workstream). This workstream is comprised of specialists working at the ERN CRANIO member healthcare centers, and includes experts of all specialisms required in cleft care.

The multidisciplinary treatment of patients with CL/P is challenging, as it starts almost immediately after birth and lasts up to adulthood. Patients with CL/ might face numerous surgical procedures; speech therapy; hearing problems; orthodontic and dentition problems; psychological support and more during childhood and adolescence [2–6].

One of the main issues with cleft care is the lack of scientific evidence for optimal treatment protocols. Consequently, many different treatment protocols have been implemented within European healthcare centers over the last decades. Therefore, the comparison of treatment outcomes between centers remains impeded. Accurate data collection is also hampered by the variation of outcome measures used in studies, which complicates the comparison of treatment outcomes between different protocols [7–10]. For the same reason, benchmarking between cleft centers is impeded. All these factors slow down innovation and progress in this field.

The lack of sound scientific evidence about optimal treatment of CL/P favors undesirable practice variation even in well-resourced countries [11]. To facilitate good comparative studies, the definition of uniform outcome measures and treatment endpoints is vital. The need to systematically register outcome measures on a European level is widely recognized within the CL/P work stream. For this purpose, ERN CRANIO will take the initiative to develop a common dataset and a European cleft registry.

To support the conceptualization of the registry, a systematic review of the literature was conducted to report on previous experiences in this field. The aim of this review is to identify the pitfalls in the development and implementation as well as factors influencing long-term success of a multidisciplinary, international registry for cleft care on a global scale. However, the findings and guidance reported in this review are meaningful for all (international) multidisciplinary collaborations seeking to register data of a pediatric patient population.

## Methods

### Search strategy and selection criteria

A systematic review was performed according to the Preferred Reporting Items for Systematic Review and Meta-Analysis (PRISMA) [12]. Members of the research team determined the search terms with a medical librarian, who developed a comprehensive search strategy. Embase, Web of Science, Medline, and ABI/INFORM Collection – ProQuest were consulted from inception up to June 2021. Online Appendix 1 of Additional file 1 contains the search strategies for each database.

Studies were included if their primary or secondary aim was to describe the design and/or the methods to develop or maintain a multidisciplinary registry that involves pediatric patients or are specifically designed for pediatric patients. As our purpose was to identify all information relevant to the development of an international database for pediatric patients, studies based on poor methodological quality or publications from before 2000 were not excluded, since these could still provide useful information on high quality registry development and design. Therefore, no method for quality assessment apart from the aforementioned exclusion criteria was used. Studies were excluded if they primarily analyzed the content of a registry (i.e., completeness or comparisons in outcomes) without assessing the design, development or maintenance of the registry itself. Studies written in any other language than English were excluded as well. In case the described registries contained information about both pediatric patients and adults, the study had to include a specific description for the pediatric population within the registry, or the differences between the registration of pediatric and adult patients had to be addressed.

Search results from all databases were merged in Endnote. Two authors independently screened for titles and abstracts according to a standardized, blinded process [13]. Full texts were reviewed independently by two authors as well. In case of disagreement, a third author was consulted. Authors were contacted by e-mail in case of any uncertainty about suitability, or in case of missing information. To find any other relevant articles, reference lists were scanned from all articles that were included after full text review.

### Data extraction and synthesis

The guidance of Popay et al. was used to establish a narrative synthesis [14]. Key themes for data extraction were first identified through thematic analysis. Two reviewers analyzed the included study, and a data extraction table was made in excel containing twenty-one headings (Table 1). The process of thematic analysis was done in an inductive and iterative manner, and several meetings were held during the process. Further, relevant information was extracted from each study by one reviewer, whereas a second reviewer cross-checked results. Data was assigned to the appropriate key theme. According to the guidance, the qualitative data collected within each key theme was then summarized. Structured narratives of each key theme are included in the results. For the extracted data that were unsuitable to be summarized into a narrative, like quantitative data or registry characteristics, tabulation was used to present a clear overview (Table 2).

**Table 1:** Collected data headings with description

Author	First author of publication
Publication year	Year article was published
Year	Year registry started
Name	Name of registry
Coverage	Regional, national or international coverage
Countries involved	Which country/countries were involved in registry
Number of patients	Number of patients enrolled in registry
Purpose	Self-defined purpose of registry
Funding	Was funding procured, who provided funding, benefits and pitfalls
Governance	Where committees present, what members
Legal	What legal basis was provided
Security	How patient data was secured and stored
Registry team	Was a registry team used, for what tasks
Design	What type of design was used for registry, what are benefits per design
Quality checks	What type of quality checks were used, benefits and pitfalls
Answer options	The types of answer options used for data entries
Data entries	Who performed data entries
Linkage	Where data linked to other databases and how
Completeness	How was data completeness improved
Participation	How was participation increased
Benchmarking	Where benchmarking reports created, with what effect

**Table 2:** Quantitative data and characteristics of reviewed registries

Anda	2017	2014	The Georgian birth registry	National	Georgia	53,236
Anda	2008	2006	Murmansk County Birth Registry (MCBR)	Regional	Murmansk	17,031
Blenstrup	2011	1993, 1980, 1994	FTDB, RLIA and IVFR	National	Denmark	NA
De Antonio	2019	2008	DM-Scope registry	National	France	2970
Deakyne-Davies	2018	2016	The PECARN registry	National	USA	894,503
Ebner	2015	NA	The ARegPKD registry	International	Germany, Turkey	NA
Ericson	1977	1973	RCM Registry and MBRS database	National	Sweden	1623
Gauvrit	2020	2009	The French Cochlear Implant Registry	National	France	5051
Gissler	2000	1995	The Finnish MBR	National	Finland	60,254
Gissler	1995	1991	The Finnish MBR	National	Finland	64,986
Hammil	2007	2008	National ICD register	National	USA	NA
Hassan	2017	2015	No name	National	Palestina	34,482
Kamper-Jorgenson	2007	2004	Childcare database	National	Denmark	1,110,973
Knox	1984	1962	National scheme and Birminghame scheme	Both regional and National	UK	NA
Lazem	2022	NA	The Iranian HUS Registry	National	Iran	NA
Mallon	2021	2016	The HBR GFOAP Registry	International	France, Burkina Faso, Ivory Coast, Senegal, DRC, Mali, Madagascar, Morocco, Cameroon, Tunisia, Togo	3349
Marazita	1992	1986	The VaCARES registry	Regional	Virginia state	10,034
Minassian	2019	2018	the pregnancy register	National	The UK	5,800,000
Nembhard	2016	1980	The WARDA registry	Regional	Western Australia	30,000
Prince	2008	2008	The ABC-register	National	The Netherlands	161
Seidlin	2017	2012	The NF registry	International	USA and 72 other Nationalities	4680
Stiller	1995	1975	The National Registry of Childhood Tumours	National	The UK	50,000
Druschel	2001	1982	Congenital Malformations Registry (CMR)	Regional	USA	NA
Shahian	2013	1989	the STS Congenital Heart Surgery Database	National	USA	NA

## Results

**Fig. 1:**
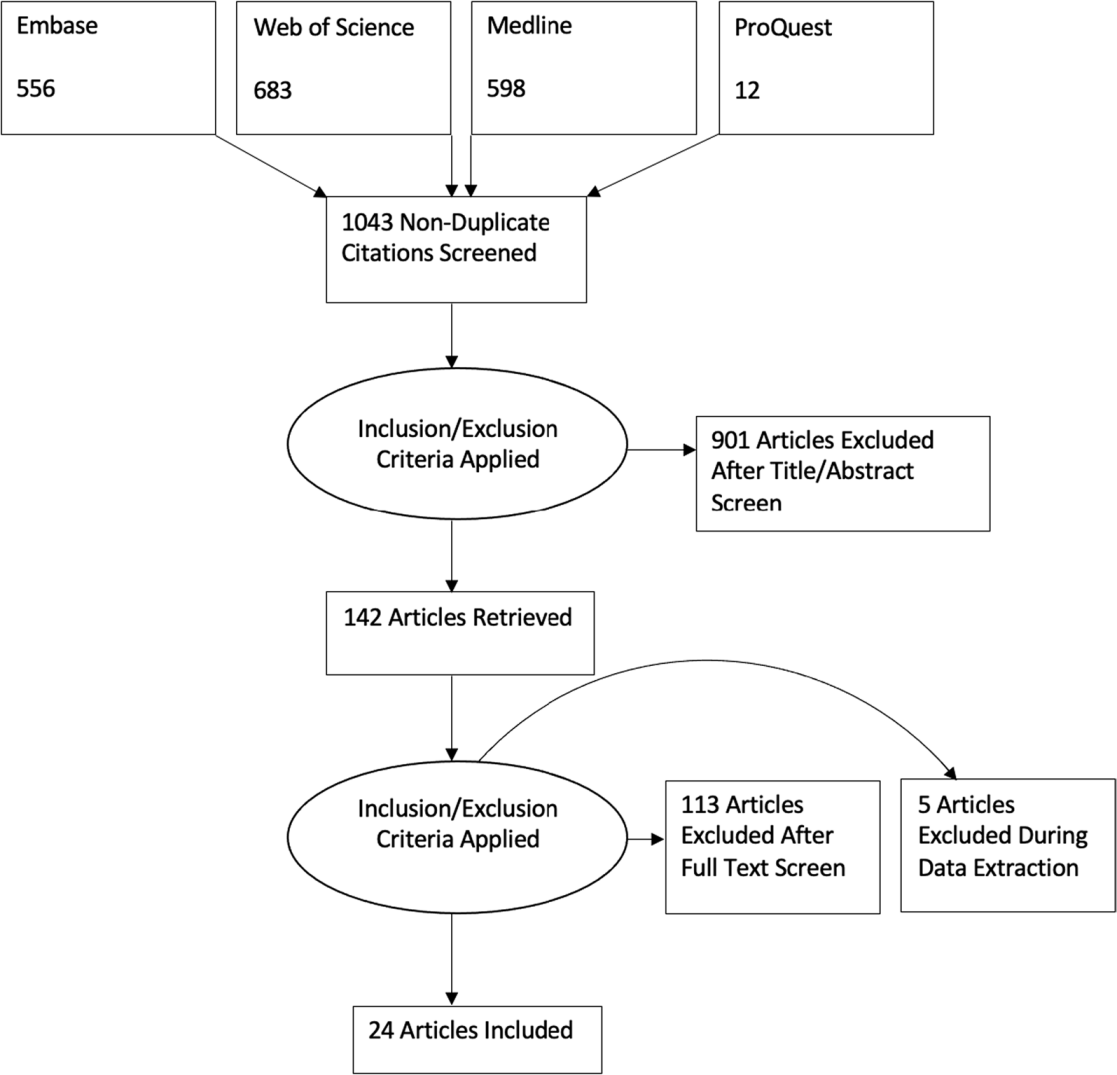
Prisma diagram

In total 1804 references were identified via Embase, Web of Science, Medline, and ABI/INFORM Collection – ProQuest up until August 2022. In total 761 duplicates were removed, and 142 publications were screened. 118 publications were excluded. Figure 1 shows the Preferred Reporting Items for Systematic Review and Meta-Analysis, PRISMA flow-chart. The complete data extraction table is available as a supplementary Excel file (Additional file 2). The headings used in the Excel file match the headings used in the article.

### Key themes

#### Purpose

The most common purpose of pediatric registries was to monitor the incidence of congenital malformations in newborns (n = 7) [15–21]. Other purposes were data-collection for performance reporting (n = 3) [17, 22, 23], research (n = 4) [17, 24–26], health monitoring (n = 2) [17, 27], and complications monitoring (n = 1) [24]. Two registries described the main purpose of creating an inter-operable multicenter framework for data collection [28, 29]. Prince et al. reported the change of a paper-based registry into electronic-based registry as the main goal of their study [30]. Other motivations for developing pediatric registries were improving international cooperation between specialized centers [28] assisting parent or caretakers of pediatric patients to access resources for support [16] Gauvrit et al. defined the purpose of the registry to report general indicator of cochlear implants in pediatric and adult patients [31].

#### Funding

Funding was essential for the development and durability of a registry [23]. Continues funding was necessary to train, hire and retain registry team members [17]. From the included studies, the majority received funding deriving from a local or national government (N = 6) [17, 20, 22, 25, 32, 33]. Other sources were nonprofit organizations [34], or via a combination of multiple sources such as government run grants, and private (industry) trusts [21, 26–28, 30–32, 35]. However, a lack of funding, or biased funding could both be detrimental to the functioning of a registry [23]. Nembhard et al. described that the change from a temporary research grant to long term government funding greatly benefitted the overall success of the registry [17].

#### Governance

In total, nine studies reported on the governance of a registry [17, 25, 28–33, 35]. Two studies noted a registry committee consisting of both patients and experts to ensure governance aligned with the purpose of the registry [28, 29]. Nembhard et al. noted that clinicians entering data felt more comfortable entering data once ownership of the registry was relinquished to government [17]. One study mentioned joint ownership of the data between two or more centers [23]. Four studies mentioned that data was owned and stored at one medical center [28, 30, 33, 35]. One study mentioned that the entire dataset was owned by the principal researcher [32], whilst another noted that data was owned by a private company funded by a patient foundation [29].

#### Legal

Seven studies noted the importance of a solid legal basis for the collection, storage, and use of data [17, 28–30, 32, 35, 36]. Three studies mentioned the registry gaining approval for implementation after consulting national authorities pertaining to medical legislation [17, 26, 28, 30, 31]. Furthermore, four studies noted that consent forms were required prior to inclusion of patients into the registry [28–30, 35]. The study by Hassan et al. mentions that implementation of the registry was performed in accordance with local jurisdiction, whilst not attaining to the medical and health research act of 2008 [32]. Lazem et al. ensured all participating centers signed data sharing and cooperation agreements as well as the national pediatric nephrology society [25].

#### Security

When storing patient data on an electronic database, multilevel security was advised when identifiable patient data were included [37]. These security measures consisted of passwords (n = 2) [17, 35], limiting access to the database to members of the registry team and only allowing de-identified patient data to be used by researchers (n = 7) [17, 22, 28, 30, 32, 34, 35]. Furthermore, in one instance data logs were used to monitor the access and behavior of users [30]. In case of a paper-based framework, secure storage, limited access, and legal identification prior to data access were most frequently used (n = 3) [21, 32, 38]. Finally, one study advised contracts regarding privacy legislation to be signed by affiliated centers prior to participation into the registry [30]. Four studies mentioned the use of servers for data storage [22, 28–30]. Most of these studies used MySql for as the management system for the database [22, 29, 30]. Hassan et al. describes the use of the DHIS2 software package [32].

#### Registry team

Labor needed for the successful running of a registry range between 0.2 full time equivalents (FTE) to 5.0 FTE [17, 22, 32, 34]. Members of registry teams reported are medical experts; principal investigators and co-researchers; and patient representatives [22, 28–30]. Responsibilities of a registry team entailed mainly data quality controls [17, 32, 34] Other responsibilities were: to improve the overall quality by updating patient case information on a regular basis, and to ensure continued completeness of data [17]. Auditing of researchers prior to relinquishing data was mentioned to be valuable to ensure data was used ethically and securely [17, 28, 33]. Furthermore, centralized registry teams produced periodic benchmark reports and structured feedback to and from participating centers. [22, 23, 30, 33]. Finally, documenting procedures and policies by the current registry team helps potential successors to work effectively after retirement of previous registry team members [17].

#### Design

Most of the studies described registries that were based on an electronic database (n = 12) [22, 23, 25–27, 29–31, 33–36] or a combination of paper-based entries and direct entries into an electronic database (n = 8) [17, 19, 20, 24, 28, 32, 38, 39]. However, two studies report the use of exclusively paper-based data entries. When classifying countries using the World Bank classification [40]. After 2005,

electronic registries used to be more frequent in high-income countries than in middle income countries and low-income countries. The popularity of electronic databases in low-income countries has increased in the past decade. However, not without issues, as Mallon et al. describe frequent power outages and poor Wi-Fi connections even after distributing laptops to participating centers [26]. Furthermore, Hassan et al. stated the implementation of an electronic database was difficult in LMIC due to the lack of existing electronic frameworks [32]. Having the option of both paper-based and electronic-based entries helped include more centers in participating with the collection of registry data [15–17, 19, 20, 24, 28, 32, 33, 38, 39]. Electronic databases were therefore an appropriate choice in countries with a solid electronic healthcare framework and had many benefits, such as direct data entries and high data quality [18, 22, 23, 27, 29, 30, 33–36]. Paper-based data entries hindered participation and had lower efficiency compared to electronic-based data entries [17]. Furthermore, initial design and conceptualization of the registry needed to be profound since later changes to the system could be simple in concept but proved difficult in execution [39]. However, changes in outcome measures after implementation of the registry should be expected in pediatric registries [33]. Shahian et al. even described regular updates of common data elements to evaluate current variables and adding new variables [33]. Finally, three studies stated that registry design flaws caused innate bias and incomplete definition of complex malformations by using low-quality data sources [16, 18, 19].

#### Quality checks

Eighteen registries discussed the implementation of data quality checks. Our thematic analysis identified four categories: manual data checks prior to the data inclusion; periodic manual data reviews; periodic auditing visitations; automatic data quality checks. Manual data checks performed [16–18, 20–23, 25–35]by the registry staff upon data-entry into the registry were the most common (n = 10) [16, 20–22, 25, 28, 31–34]. Periodic auditing visitations were described in three studies [21, 28, 32]. Alternatives mentioned were automated data quality checks, consisting of plausible ranges, mandatory answer options and duplicate removal (n = 4) [22, 28, 33, 35]. Periodic manual data reviews were also used in two examples [23, 26, 32]. Furthermore, in one registry, after initial data entry, a summary was presented to the participant to confirm the correctness of the data [30]. Regarding internal validity of data in the registry, erroneous or inaccurate data entries should be anticipated and prevented [15–18, 21, 34]. On top of that, several studies advised training of the medical staff at the affiliated centers [21, 26, 28, 32].

#### Answer options

Concerning answer options, multiple designs were suggested. The use of some form of standardized answer options (SAO) was mentioned in eighteen studies[15–19, 21–23, 25, 26, 28–30, 33–35, 38, 39]. Several variations were described, of which tick-box answers, and international classification of Disease (ICD) codes [41, 42] were commonly used [16, 18, 21, 32, 38].

Three studies reported the use of SAO exclusively, while eight studies implemented both questions with free text answer options as well as SAO. Facilitating both options allowed for more profound and descriptive information to be captured [15, 17–19, 22, 25, 26, 28–30, 33, 34, 39]. In registries concerning more complex multi-malformations or diseases with phenotypical variety, this strategy was reported to be especially useful [35]. As a supplement to written descriptions, the inclusion of drawings, photographs or x-photographs were of added value [18]. Changing the framing of questions substantially changed the outcome of the questions and should thus be done cautiously according to Minassian et al. [27].

#### Data-entries

Various data-entry options were discussed in the included studies. Most registries (n = 9 reported that data was entered exclusively by clinicians or medical professionals [18, 20, 21, 23, 25, 26, 28, 30–32, 34, 39]. Two studies reported the data entries were performed by a dedicated registry team [17, 28]. Seidlin et al. reported that data was entered solely by patients [29]. In the latter case, continues monitoring of data entries was advised, since disease specific surveys can contain questions prone to misunderstanding. To increase correct data entries, patients were provided with a glossary and photographs of the most major manifestations of the disease [29]. Moreover, Hassan et al. concluded that the amount of data that should be entered has to be considered as well: extensive lists of variables that were not part of routine documentation prior to the registry were viewed as tedious by medical professionals. This caused missing data entries and even low-quality data entries in the registry [32].

#### Linkage

In total, eleven studies mentioned some form of linkage. Most of these registries allowed for linkage to more than one database or population-based registry, to increase data completeness (N = 8) [17, 19, 20, 24, 27, 33, 36, 38]. Designing the registry with personal identifiers was advised in three studies [19, 20, 34], since this allows for linkage to other registries or population-based databases. Linkage of databases was described as a powerful tool to improve data completeness [15, 17, 19, 20, 24, 27, 33, 36, 38].

#### Completeness

Completeness of data was limited by missing data; incomplete data; failure to complete data after linkage or failure to follow-up during data quality checks (n = 6) [16, 21, 22, 24, 27, 28]. Six registries contacted and implored participating centers to correct dubious data entries and false entries, or complete missing entries [20, 22, 28, 32, 33, 35]. Furthermore, to promote participating centers to enter high quality and complete data, two studies used immediate feedback forms after data entries to allow for instantaneous correction by the participants [30, 33]. Completion was improved via routine linkage to other databases or registries in three studies, [17, 18, 27] or via active data ascertainment by a dedicated research team [17]. In case of repeated incomplete or dubious data entries, one registry used a regional coordinator to provide support to the participating center [35]. In case of patient entered data, frequent reminders to complete or update personal data increases data entries were sent to patients [29]. Finally, Mallon et al. report that regular site visits to the participating centers helped increase data completeness [26].

#### Participation

Findings regarding participation were noted in sixteen studies. The thematic analysis showed that participation was influenced by different factors. First, the affiliated centers must feel that the active participation positively affects health outcomes, without causing a disproportionate increase in workload [32]. To ensure involvement of affiliated centers, two studies organized periodic meetings to inform on the functioning and future perspectives of the registry at the center’s site [20, 39] Nembhard et al. suggested active feedback ascertainment [17]. This appeared to improve participation of medical professionals and patients, as well as data quality. Financial reimbursements for completing data entries were mentioned to improve overall motivation of clinicians to participate [34]. Mallon et al. reported financially reimbursing participating centers after more than fifty new case entries to compensate for time [26]. Lastly, the study by Seidlin found a correlation between patient entered data entries and a yearly reminder e-mail to complete data entries [29].

#### Benchmarking

The use of periodic benchmarking reports was mentioned in five studies [22, 23, 30, 32, 33]. The advantages reported were the allowance for continued comparisons between participating centers, for caveats in performance to be addressed and to improve resource utilization [22, 23, 30, 33, 39]. Furthermore, benchmarking or performance reports allowed participating centers to routinely review data in their respective database [22, 25]. In one study, participating centers could voluntarily choose to share personal benchmarking reports with patients or other participating centers. This transparency allowed patients to make better decisions regarding healthcare providers [33].

## Discussion

In this systematic review, through narrative analysis, the key themes for developing a successful multidisciplinary registry for pediatric patients have been identified. While the goal of our research was to provide information for the design of the ERN-CRANIO initiative of a new European registry for patients with a cleft. However, the information that is provided in this review can be applied to a wider pediatric patient group and provides support in the development of other registries.

### Summary of key themes

**Fig. 2:**
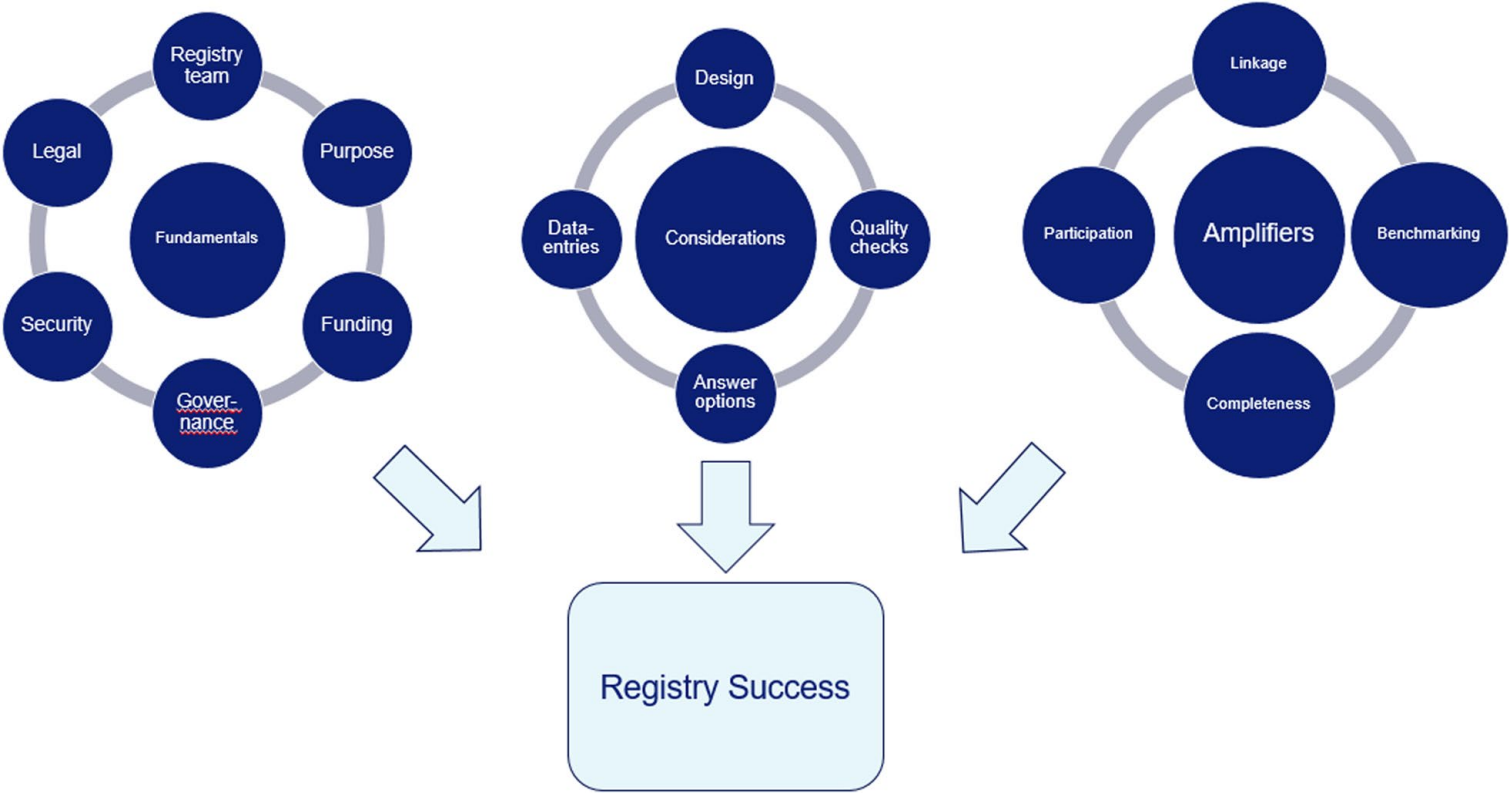
Key-themes required for a successful international multidisciplinary registry for pediatric patients

This systematic review successfully identified the key themes necessary for the long-term success of an international, multidisciplinary registry for pediatric patients. All key themes are important challenges on their own. In order to provide a hands-on, stepwise approach for the development of a registry, the key themes have been subdivided in three categories: “fundamentals”, “considerations”, and “amplifiers”. This is visualized in Fig. 2. The key themes included in the “fundamentals” category needs to be fully developed before the conceptualization faze of a registry. If one or more of these themes is not completely formulated, registry success will be unlikely. The themes categorized in the “considerations” subgroup are concepts that may differ depending on the purpose of the registry. Therefore, these themes require careful consideration prior to implementation and during the running of the registry. Finally, the themes discussed in “amplifiers” are important to increase the quality of the registry. The better the key themes categorized in the “amplifiers” subgroup are implemented, the higher the data quality and completeness will be. The themes per category will be discussed in more detail in the following paragraph in the form of a written guidance, a visual guidance useful during conceptualization of an international pediatric registry is available in the online appendix 2 of Additional file 1.

### Guidance of key themes

#### Fundamentals

The purpose of a registry needs be clear for all participating parties and predetermined prior to the implementation of a registry [43]. This purpose can be diverse, such as health monitoring, quality control or scientific research. Currently, registries are getting used more in research for cost-effective data collection. Furthermore, registries are becoming more popular when flexible research design is needed over randomized controlled trials [43–47]. To warrant durability and functionality of a registry, unbiased funding is required [43]. Funding can be procured via private-sector funding, charities, and non-profit organizations such as the World health organization (WHO) as well as via the government [47]. However, a combination of different sources is not uncommon and expected to become more frequent in the future [43]. Long term funding is especially important for the long-term success of a registry. The design, size and purpose of the registry all influence the amount of funding needed to succeed [43]. Therefore, it is essential to include funding in the entire process of creation and implementation of a registry. To ensure the success of a registry governance is needed. Governance can provide guidance in the form of committees consisting of medical specialists, researchers, and patient representatives. These committees can provide high-level decision making, relating to purpose, funding, and data relinquishing [43]. Furthermore, the WHO suggests publicly stating ownership and governance structure with regards to a registry. (Source) When a registry is operating on an international scale, local data ownership is preferred, due to the legal implications associated with patient data. Privacy laws are strict and can be nation or center specific, thus international ownership of data can prove very difficult practice [48]. Data custody without data ownership can therefore be an appropriate solution to this problem [49]. Furthermore, it is important to consider which data are to be entered in the registry. Variables exceeding standard practice, at least in Europe are subject to more extensive and strict requirements [50]. To guarantee security of patient data, data access should be limited via multilevel security. Limiting full data access to registry to a data committee ensures patient privacy is preserved. Passwords and user logs provide additional protection. Further data protection can be realized by creating data logs and user behavior screening. Numerous free to use software for database provide such services [51–53]. Furthermore, for local storage, secure servers hosted by the medical centers themselves are recommended. However, in the future, this could change via the General Data Protection Regulation of the European Union. The GDPR ensures that data protection throughout Europe is compliant to the highest standards [54]. Possibly, this could facilitate centralized data ownership in Europe in the future. Another key factor for long-term success of a registry is a registry team. Such teams can be used for manual quality control of entered data. A registry team can create periodic benchmarking reports and consistently perform visitations to participating centers and audit researchers and perform active data ascertainment. Like other registry committees, registry teams can consist of different members, such as medical experts, researchers, and patient representatives. The required full-time equivalents (FTE) required by the registry team to run a successful registry is highly depended on the purpose and size of the registry [43].

#### Considerations

**The design** of a registry is dependent on the purpose for which the registry is created. When designing a registry, it is important to consider what operating framework is best suited for the purpose, the patient population, and the area in which the registry operates This systematic review shows that an electronic based registry is preferred over a paper-based registry provided local ICT facilities allow to do so. An electronic registry allows for higher data quality, higher participation, and immediate data entries. Furthermore, in an international setting, electronic databases allow for instantaneous global functionality. However, it is important to realize that within Europe, internet access and digital skills are not equally distributed throughout the continent [55]. Additionally, even in countries with an electronic infrastructure, health centers can still rely on paper-based records [56]. When multidisciplinary outcome measures are to be captured, the framework of a registry should be accommodating to change, since different specialties might change or expand on the outcome measures. This especially holds true for pediatric registries, since it requires long term follow-up of patients. For instance, tools used to assess patients to change or improve over the years, thereby changing the collected outcome measures over time. However, changing outcome measures should ideally be kept to a minimum since this can be easy in concept but prove difficult in execution. For instance, new data additions might require changes in the framework, training personnel on sight, adjustments to registry protocols and amendments at the medical ethics board. This also results in more financial recourses being used [43]. To ensure the data included in the registry are of high quality and suited for the predetermined purpose of the registry, quality checks need to be implemented. Automated and manual data checks prior to inclusion of data into the registry, periodic manual data checks, periodic auditing visitations and automated data checks are all advised to guarantee continued high data quality. The training of staff at the participating centers is also a viable way to ensure data quality is high. Finally, it is important to consider what answer options are best suited for the registry. By using free-text answer options, SAO, and additional data entries such as photographs, a more profound description of malformations can be achieved. To ensure participants remain motivated to put in the effort of completing data entries over longer time periods, the effort to complete data entries should be reasonable and in line with de purpose of the registry. Therefore, it is important to distinguish between a core data set, ideally consisting of the minimum requirements for achieving the purpose, and additional more extensive data entries useful for further research initiatives. Thus, the purpose of the registry equally dictates the number of variables included in the registry. The WHO suggests appointing a “responsible registrant” to perform and check data-entries to ensure high-quality data is continuously uploaded in the registry [47]. Furthermore, the complexity of clinical outcome measures being captured influences the quality checks required to examine the data entries [57].

#### Amplifiers

The use of personal identifiers is recommended within a local server system to ensure the possibility of linkage to other local or national databases and registries. This can help increase the overall completeness of the collected data in the registry. High completeness of data is mentioned as an indicator of registry success [58–60]. Using entries by both clinicians and patients improves the overall understanding of malformations. However, in case of patient entered data, careful monitoring is necessary to prevent erroneous data to be entered into the registry. This requires a dedicated team [43]. Furthermore, extensive lists of variables are viewed as tedious and are at risk of not being entered correctly. This not only influences data quality since this negatively influences participation of medical centers. However, by organizing periodic meetings to inform on projects and research efforts arising from the data entered in the registry, participation can be increased. Furthermore, participation was reported to increase when active feedback ascertainment was organized by the registry team. Participation can also be increases by allowing participating centers to co-author research initiatives [60, 61]. Lastly, compensating participating centers after completing a pre-determined amount of complete data entries can improve participation [26]. Another frequently noted way of improving completeness and participation was via benchmarking reports. Benchmarking reports are a useful tool to allow for inter-center comparison in performance and help improve participation and health outcomes for patients. Lastly, if possible, financial reimbursements for participating centers after complete and correct data entries are proven to be effective in increasing participation.

### Relevance to ERN CRANIO and other research

The ERN CRANIO initiative has recognized the need for a successful, long-term registry to improve cleft care on an international level. A successful registry can facilitate international comparison between centers, improve international research initiatives, provide data for international benchmarking and the improvement of overall healthcare quality [58–60]. Additionally, registries are noted to be very suitable for surveillance of rare diseases and for performing studies relating to conditions with complex treatment patterns [57]. An example of such an epidemiological registry in Europe is EUROCAT [62]. This initiative surveys, collects and analyses data on congenital anomalies across populations in Europe since 1979, proving the need and use for such European-wide registries. The ERN CRANIO CL/P registry will also collect epidemiological data; however, it will mainly be focused on collecting comparable clinical and patient reported outcome measures to assess treatment outcomes between centers. Lastly, prospective registries have been noted as cost-effective alternatives for clinical trials [60, 61]. This fact has also been noted by the World Health Organization (WHO), which is currently supporting and launching seventeen distinct trial registries [47]. This systematic review identified the key themes that influence the overall success and longevity of an international multidisciplinary registry for pediatric patients. This information is not only useful in relation to the aims of the ERN CRANIO initiative but can be used by anyone seeking guidance in the development of a registry.

### Strengths and limitations

Due to the nature of narrative analysis, the types of included studies varied. The types of publications included: registry reviews, descriptive articles, and data analysis reports. Additionally, publications spanned over forty years. Furthermore, the development of the thematic data extraction set is possibly affected by researcher bias. However, by including a second researcher for the cross-referencing of the publications and the thematic data extraction set it was attempted to mitigate this bias. By including non-international registries into the systematic review, the specificity of the experience of developing such registries may have been diluted. However, by including non-international registries, more overall experience including strengths and pitfalls for successful registries could be analyzed. The inclusion criterion of registries needing to be developed for pediatric patients was not something that was mentioned in the reviewed literature as being distinctly different from developing any other registries. However, we postulate that pediatric registries tend have more assessments over a longer period of time that in adult registries and could therefore be, generally, more complex than adult registries. A strength of this review is that by systematic narrative analysis a scientifically sound basis for the development of a pediatric multidisciplinary registry is provided.

## Conclusion

This systematic review provides a scientific basis that aids the ERN initiative in creating a European-wide registry for collecting outcome measures relating to the multidisciplinary treatment of patients with cleft palate. Main pitfalls negating and key themes relating to long-term registry success have been identified via narrative analysis. This review can be useful to any initiative seeking to develop a registry. Furthermore, this review provides methodological tools that can provide help in the development of a registry framework on a wider international and pediatric basis. A future descriptive study reporting on the practical experiences during the development and implementation of the registry could complement this review and provide profound insights for other registry initiatives.

### Supplementary Information

**Additional file 1.** Appendix.

**Additional file 2.** Standardized basic data infromation supplemented with disease specific data and bio material.

## Data Availability

All data generated or analyzed during this study are included in this article and its additional information files.

## Abbreviations

ERN-CRANIO: European Reference Network for craniofacial anomalies and ear, nose and throat disorders
CL/P: Cleft lip and palate
