# Supplementary material for: Guidance to develop a multidisciplinary, international, pediatric registry: a systematic review
Source: Orphanet J Rare Dis. 2023 Sep 21;18:296. doi: 10.1186/s13023-023-02901-4 (PMC10512647; doi:10.1186/s13023-023-02901-4)

## Appendix

Appendix 1: *Search strategy*

#### Appendix 1:

| **Database searched** | **Platform** | **Years of coverage** | **Records** | **Records after duplicates removed** |
| --- | --- | --- | --- | --- |
| Embase | Embase.com | 1971 - Present | 556 | 542 |
| Medline ALL | Ovid | 1946 - Present | 598 | 171 |
| Web of Science Core Collection* | Web of Knowledge | 1975 - Present | 638 | 325 |
| One Business | ProQuest |  | 12 | 5 |
| **Total** | | | **1804** | **1043** |

*Science Citation Index Expanded (1975-present) ; Social Sciences Citation Index (1975-present) ; Arts & Humanities Citation Index (1975-present) ; Conference Proceedings Citation Index- Science (1990-present) ; Conference Proceedings Citation Index- Social Science & Humanities (1990-present) ; Emerging Sources Citation Index (2005-present)

*No other database limits were used than those specified in the search strategies below*

**Embase**

((('register'/de/mj) AND ('benchmarking'/exp OR 'quality control'/de)) OR (((register OR registers OR registry OR registries OR EMR-based OR administrative*-database* OR record-system*) NEAR/15 (quality-control* OR quality-audit* OR ranking OR benchmark* OR content* OR completeness OR collection-method* OR data-amount* OR data-qualit*))):ab,ti,kw) **AND** (child/exp OR adolescent/exp OR adolescence/exp OR 'child behavior'/de OR 'child parent relation'/de OR pediatrics/exp OR childhood/exp OR 'child nutrition'/de OR 'infant nutrition'/exp OR 'child welfare'/de OR 'child abuse'/de OR 'child advocacy'/de OR 'child development'/de OR 'child growth'/de OR 'child health'/de OR 'child health care'/exp OR 'child care'/exp OR 'childhood disease'/exp OR 'child death'/de OR 'child psychiatry'/de OR 'child psychology'/de OR 'pediatric ward'/de OR 'pediatric hospital'/de OR 'pediatric anesthesia'/de OR 'pediatric intensive care unit'/de OR 'neonatal intensive care unit'/de OR 'prematurity'/de OR (adolescen* OR preadolescen* OR infan* OR newborn* OR (new NEXT/1 born*) OR baby OR babies OR neonat* OR prematur* OR pre-matur* OR child* OR kid OR kids OR toddler* OR teen* OR boy* OR girl* OR minors OR underag* OR (under NEXT/1 (age* OR aging OR ageing)) OR juvenil* OR youth* OR kindergar* OR puber* OR pubescen* OR prepubescen* OR prepubert* OR pediatric* OR paediatric* OR school* OR preschool* OR highschool* OR suckling* OR PICU OR NICU OR PICUs OR NICUs):ab,ti,kw) *NOT ([Conference Abstract]/lim) NOT ([animals]/lim NOT [humans]/lim) AND [ENGLISH]/lim*

**Medline**

(((*Registries/) AND (Benchmarking/ OR Quality Control/)) OR (((register OR registers OR registry OR registries OR EMR-based OR administrative*-database* OR record-system*) ADJ15 (quality-control* OR quality-audit* OR ranking OR benchmark* OR content* OR completeness OR collection-method* OR data-amount* OR data-qualit*))).ab,ti,kf.) **AND** (exp Child/ OR exp Infant/ OR exp Adolescent/ OR exp "Child Behavior"/ OR exp "Parent Child Relations"/ OR exp "Pediatrics"/ OR "Child Nutrition Sciences"/ OR "Infant nutritional physiological phenomena"/ OR exp "Child Welfare"/ OR "Child Development"/ OR exp "Child Health Services"/ OR exp "Child Care"/ OR "Child Rearing"/ OR exp "Child development Disorders, Pervasive"/ OR "Child Psychiatry"/ OR "Child Psychology"/ OR "Hospitals, Pediatric"/ OR exp "Intensive Care Units, Pediatric"/ OR (adolescen* OR infan* OR newborn* OR (new ADJ born*) OR baby OR babies OR neonat* OR prematur* OR pre-matur* OR child* OR kid OR kids OR toddler* OR teen* OR boy* OR girl* OR minors OR underag* OR (under ADJ1 (age* OR aging OR ageing)) OR juvenil* OR youth* OR kindergar* OR puber* OR pubescen* OR prepubescen* OR prepubert* OR pediatric* OR paediatric* OR school* OR preschool* OR highschool* OR suckling* OR PICU OR NICU OR PICUs OR NICUs).ab,ti,kf.) *NOT (news OR congres* OR abstract* OR book* OR chapter* OR dissertation abstract*).pt. NOT (exp animals/ NOT humans/) AND english.la.*

**Web of Science**

TS=(((((register OR registers OR registry OR registries OR EMR-based OR administrative*-database* OR record-system*) NEAR/15 (quality-control* OR quality-audit* OR ranking OR benchmark* OR content* OR completeness OR collection-method* OR data-amount* OR data-qualit*)))) **AND** ((adolescen* OR preadolescen* OR infan* OR newborn* OR (new NEAR/1 born*) OR baby OR babies OR neonat* OR prematur* OR pre-matur* OR child* OR kid OR kids OR toddler* OR teen* OR boy* OR girl* OR minors OR underag* OR (under NEAR/1 (age* OR aging OR ageing)) OR juvenil* OR youth* OR kindergar* OR puber* OR pubescen* OR prepubescen* OR prepubert* OR pediatric* OR paediatric* OR school* OR preschool* OR highschool* OR suckling* OR PICU OR NICU OR PICUs OR NICUs)) NOT ((animal* OR rat OR rats OR mouse OR mice OR murine OR dog OR dogs OR canine OR cat OR cats OR feline OR rabbit OR cow OR cows OR bovine OR rodent* OR sheep OR ovine OR pig OR swine OR porcine OR veterinar* OR chick* OR zebrafish* OR baboon* OR nonhuman* OR primate* OR cattle* OR goose OR geese OR duck OR macaque* OR avian* OR bird* OR fish*) NOT (human* OR patient* OR women OR woman OR men OR man))) NOT DT=(Meeting Abstract OR Meeting Summary) AND LA=(English)

**Abi/Inform (applied filters: scholarly journals and working papers)**

AB,TI(((((register OR registers OR registry OR registries OR EMR-based OR administrative*-database* OR record-system*) **N/**15 (quality-control* OR quality-audit* OR ranking OR benchmark* OR content* OR completeness OR collection-method* OR data-amount* OR data-qualit*)))) **AND** ((adolescen* OR infan* OR newborn* OR (new N/1 born*) OR baby OR babies OR neonat* OR prematur* OR pre-matur* OR child* OR kid OR kids OR toddler* OR teen* OR boy* OR girl* OR minors OR underag* OR (under N/1 (age* OR aging OR ageing)) OR juvenil* OR youth* OR kindergar* OR puber* OR pubescen* OR prepubescen* OR prepubert* OR pediatric* OR paediatric* OR school* OR preschool* OR highschool* OR suckling* OR PICU OR NICU OR PICUs OR NICUs))) NOT PT (news OR comment* OR editorial* OR congres* OR abstract* OR book* OR chapter* OR dissertation abstract*) AND LA(english)

Appendix 2: *Please reference the PDF-file: “visual guidance for registry.pdf” for a high-definition version of the visual guidance.*


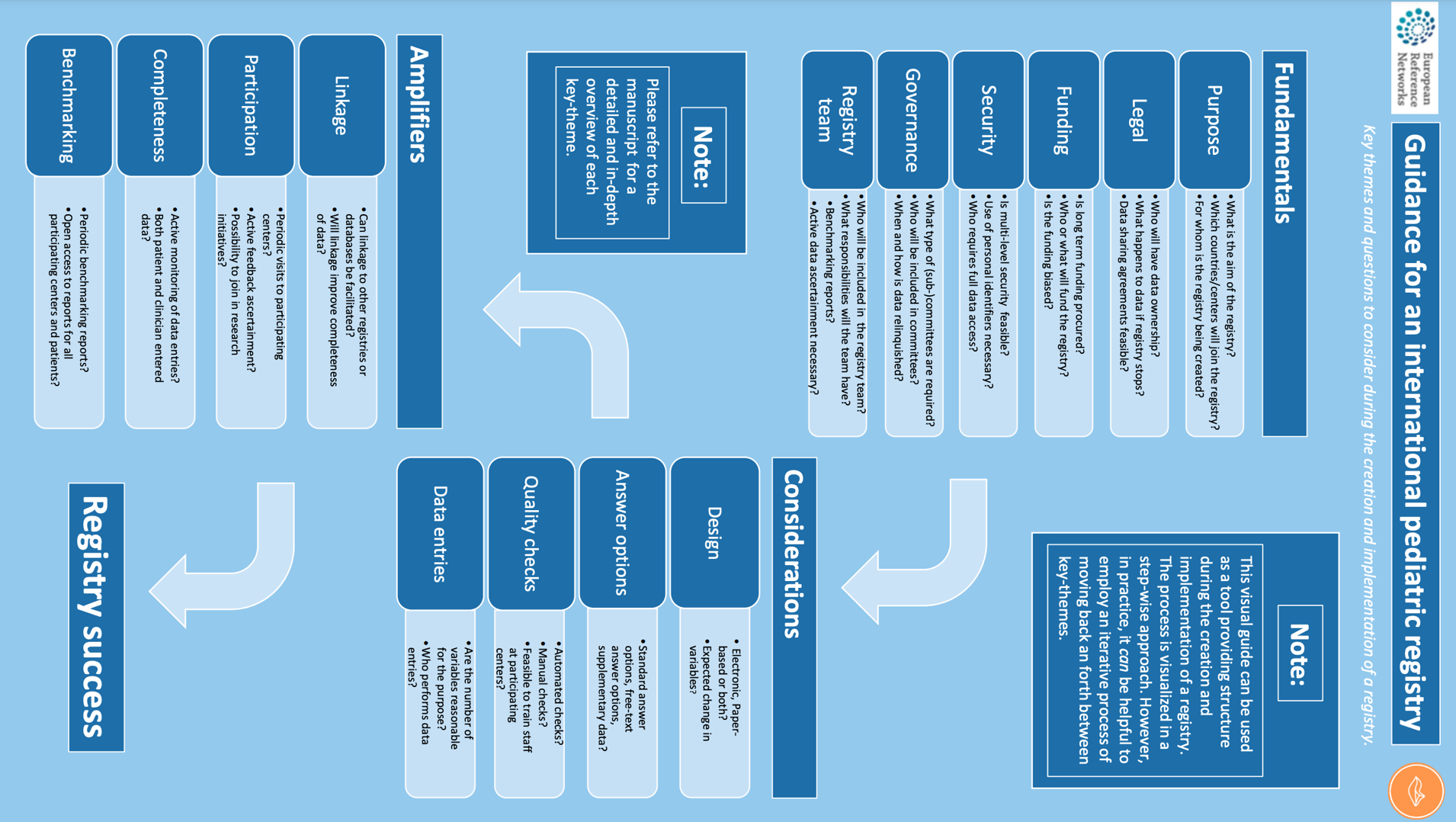

Supplement: Supplementary file 1 — Additional file 1. Appendix. [file 13023_2023_2901_MOESM1_ESM.docx]
